# A three-dimensional multivariate image processing technique for the analysis of FTIR spectroscopic images of multiple tissue sections

**DOI:** 10.1186/1471-2342-6-12

**Published:** 2006-10-03

**Authors:** Bayden R Wood, Keith R Bambery, Corey J Evans, Michael A Quinn, Don McNaughton

**Affiliations:** 1 Centre for Biospectroscopy and School of Chemistry, Monash University, 3800 Victoria, Australia; 2 Department of Chemistry, University of Leicester, Leicester, LE1 7RH, UK; 3 Department of Obstetrics and Gynaecology, Royal Women's Hospital, Grattan St. Parkville, 3052, Victoria, Australia Sciences, Monash University, 3800 Victoria, Australia

## Abstract

**Background:**

Three-dimensional (3D) multivariate Fourier Transform Infrared (FTIR) image maps of tissue sections are presented. A villoglandular adenocarcinoma from a cervical biopsy with a number of interesting anatomical features was used as a model system to demonstrate the efficacy of the technique.

**Methods:**

Four FTIR images recorded using a focal plane array detector of adjacent tissue sections were stitched together using a MATLAB^® ^routine and placed in a single data matrix for multivariate analysis using Cytospec™. Unsupervised Hierarchical Cluster Analysis (UHCA) was performed simultaneously on all 4 sections and 4 clusters plotted. The four UHCA maps were then stacked together and interpolated with a box function using SCIRun software.

**Results:**

The resultant 3D-images can be rotated in three-dimensions, sliced and made semi-transparent to view the internal structure of the tissue block. A number of anatomical and histopathological features including connective tissue, red blood cells, inflammatory exudate and glandular cells could be identified in the cluster maps and correlated with Hematoxylin & Eosin stained sections. The mean extracted spectra from individual clusters provide macromolecular information on tissue components.

**Conclusion:**

3D-multivariate imaging provides a new avenue to study the shape and penetration of important anatomical and histopathological features based on the underlying macromolecular chemistry and therefore has clear potential in biology and medicine.

## Background

The ability to generate and manipulate three-dimensional (3D) images of body parts or tissue sections is extremely useful in determining the extent and penetration of disease or tissue degeneration. Conventional ways of generating such 3D images are Computerized Tomography (CT), Positron Emission Tomography (PET), Magnetic Resonance Imaging (MRI) and 3D ultrasound. X-ray based techniques are becoming more useful with the increased contrast available by coupling the technique with synchrotron radiation and using phase contrast and diffraction enhanced imaging. These techniques do not supply information on the macromolecular composition in the image contrast, whereas spectroscopy based techniques do, and hence 3D IR imaging would provide a useful and novel alternative with the advantage of image contrast based directly on the underlying macromolecular composition. The lack of penetration of mid IR radiation into tissue precludes real time imaging of whole samples but an alternative is to build a composite from 2D images of adjacent sections of tissue thus providing a method to gauge the extent and penetration of disease, which may be of clinical value. This has the advantage of not requiring a chemical or immunological staining protocol to provide biochemical information. High-speed low-cost computers, in combination with infrared imaging instruments based on Focal Plane Array (FPA) detectors, allow the image acquisition and reconstruction to be achieved within a reasonable time frame.

The adaptation of multi-channel infrared array detectors from military hardware to FTIR microscopes in the early 1990s resulted in new methodologies to investigate the macromolecular architecture of cells in tissue sections [1]. The new generation of FPA and more recently linear array detectors are capable of recording thousands of spectra in rapid time. Each pixel is essentially a digital hyper-spectral data cube containing absorbance, wavenumber and ***x***,***y ***spatial coordinates. Univariate or chemical maps can be plotted based on peak height, integrated areas under specific bands or band ratios. While these maps provide spatial information on the distribution and relative concentration of the major macromolecules they are not useful in correlating anatomical and histopathological features with corresponding spectral profiles [2]. Multivariate imaging techniques including Unsupervised Hierarchical Cluster Analysis (UHCA) [2-9], K-means clustering [8,10], Principal Components Analysis (PCA) [11], Linear Discriminant Analysis [12], Fuzzy C-means clustering [8,13] and neural networks [11] have proven to be invaluable in the identification of spectral groups or "clusters" which can be directly compared to stained tissue sections. In multivariate methods, the information of the entire spectrum can be utilized for the analysis. The first part of the analysis requires a distance matrix to be calculated. This can be achieved using a number of different algorithms including D-values (Pearson's correlation coefficient), Euclidean distances, normalized Euclidean distances, Euclidean squared distances and City Block all of which are available in the Cytospec™ software package [14] and appear to produce similar cluster maps although the time taken for each method can vary. We used the D-values method because this is a well-established linear regression method that is suited to relative concentration data. One disadvantage of this algorithm is that it is computationally more demanding than others; therefore more time is required for the distance matrix calculation.

In cluster analysis a measure of similarity is established for each class of related spectra and a mean characteristic spectrum can be extracted for each class. In the final step, all spectra in a cluster are assigned the same color. In the false color maps, the assigned color for each spectral cluster is displayed at the coordinates at which each data cube was collected. The mean spectrum of a cluster represents all spectra in a cluster and can be used for the interpretation of the chemical or biochemical differences between clusters. There are also a variety of algorithms to select from to perform cluster analysis, including Ward's algorithm, which we employ because it minimizes the heterogeneity of the clusters.

The high correlation of spectral clusters with anatomical and histopathological features has been conclusively demonstrated for a number of different tissue types including cervical [2,3], breast [10,15], liver [4,7], brain [5], mouth [6], intestine [8,16], skin [17], bone [18,19], cornea [20] and prostate [21]. Hitherto FTIR multivariate imaging has been mainly restricted to the generation of 2D cluster maps. The exception is Mendelsohn and coworkers [22] who constructed a 3D univariate map of cortical bone based on peak ratios from serial two-dimensional sections. By interfacing two types of software namely Cytospec [14] and SCIRun [23] and writing a simple "stitching" algorithm we are able to generate 3D multivariate cluster maps from multiple tissue sections. The ability to visualize 3D FTIR cluster maps provides a new avenue to assess variation in multiple tissue sections and to determine the penetration of histopathological structures based on the underlying macromolecular structure of the diseased tissue.

## Methods

Following approval from the Royal Women's Hospital Research and Human Research Ethics Committees and the Monash University Standing Committee on Ethics in Research Involving Humans, written, informed preoperative consent was obtained from the patient and a cervical tissue sample exhibiting villoglandular adenocarcinoma was then obtained by cone biopsy. The tissue sample was then embedded in a paraffin block and sliced by microtome into 4 μm sections. One group of four sections was mounted on glass slides and stained with the routine histopathology stain Hematoxylin and Eosin (H&E) for light microscope examination. Hematoxylin has an affinity with nucleic acids and Eosin has an affinity for the cellular cytoplasm. An adjacent group of four sections was deparaffinized, mounted on Kevley™ "low e" IR reflective microscope slides and imaged with a Varian Stingray FTIR microscope system equipped with a 64 × 64 pixel HgCdTe liquid nitrogen cooled FPA with a 15× Cassegrain objective. FTIR hyper-spectral data images were recorded in the range 4000-950 cm^-1 ^at 6 cm^-1 ^resolution and with 16 scans co-added. For each of the four sections, step-motion control of the microscope stage was used to construct a 16 tile (4 × 4) FTIR image mosaic from FPA recordings collected as 16 pixel aggregates. Thus the spatial resolution obtained is approximately 22 μm per pixel aggregate. Each FTIR image was therefore 2.0 mm^2 ^in area and with the four 4 μm thick adjacent sections giving a total sampled volume of 1,400 × 1,400 × 16 μm. A spatial resolution of 22 μm per pixel was used as this provided FTIR images that covered an area of tissue large enough to encompass several examples of anatomically different tissue types.

Using a MATLAB^® ^routine developed by our group, the four FTIR images were stitched together side by side (or "unfolded") to give a single large 2D image frame (see additional file 1 "cyto4fs.m" a program for stitching multiple tissue sections together for use in Cytospec™ spectroscopic software). The absorbance was integrated over a large spectral region (1750-950 cm^-1^) to assess sample thickness using a routine in Cytospec™. This avoids inaccuracies with too thin samples with low absorbance or too high absorbance that result in non-linear detector response. A spectrum is rejected if the determined integration value is higher or lower than a pre-defined threshold (1500 and 50 arbitrary units). Spectra that passed the thickness quality test were converted to second derivative spectra using a Savitsky-Golay algorithm (13 smoothing points). UHCA (*D*-values, Ward's algorithm) was performed to generate 4 clusters from second derivative spectra over the 1272-950 cm^-1 ^spectral window. The resultant cluster map was then reorganized (or "back folded") into the four individual 2D cluster maps, each map corresponding to one of the FTIR images. The four cluster maps were saved in an image file format with a unique false color assigned to each cluster and then aligned or "registered" as separate floating layers of a single image in the GIMP [24] image-processing program. This registration step is necessary as the sample orientation was not identical in both rotation and translation on each of the four acquired 2D mosaic images. Proper pixel correspondence from one image to another was easily achieved using this manual approach given the small number of image layers. The registered layers constituted a best fit because some slightly unequal distortion of the tissue matrix was observed presumably caused by the sectioning and preparation processes. For this 3D imaging technique, special care must be taken to ensure the sections are not stretched or distorted when deposited onto the slides.

The SCIRun [23] software suite provides a graphical user interface for rapid development of "networks" of instruction routines for the stacking and rendering of the input data (see additional file 2 "SCIRun adenocarcinoma.net" for the 3D image processing program modules and configuration parameters for SCIRun). The registered images were loaded into SCIRun as a set of indexed integer values (1 to 4 corresponding to each cluster) and then "stacked" into a scalar volume field of cluster values from which the 3D cluster maps were rendered.

**Figure 1:**
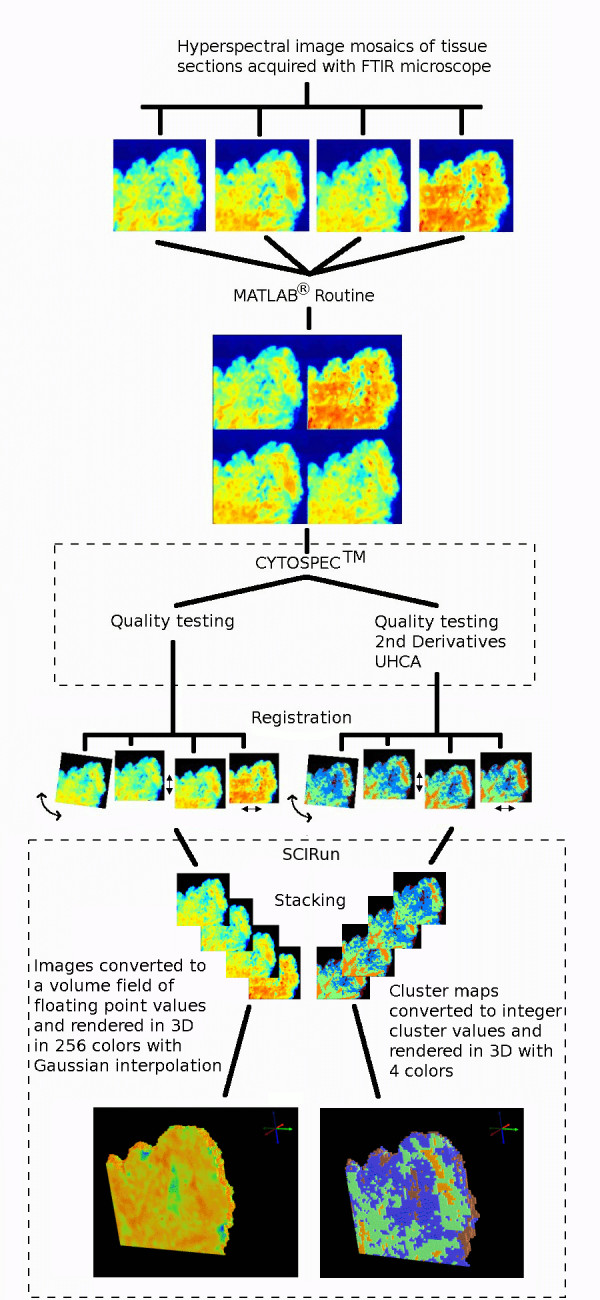
Schematic showing steps in the construction of 3D FTIR multivariate images.

3D univariate chemical maps depicting a single spectral feature were also generated. The spectra were vector normalized over the 1800-950 cm^-1 ^range and then integrated under the absorbance band of interest using a trapezoidal baseline function in Cytospec™. The 3D univariate maps were rendered from a scalar volume field of absorbance values generated from 2D FTIR images stacked in SCIRun. The 3D univariate maps were plotted with a 256 rainbow color palette using Gaussian interpolation between the data grid points to produce a smoothly varying color field. The 3D cluster maps, on the other hand, were plotted in a palette of only 4 false colors and box interpolated, with one false color corresponding to each cluster. Figure 1 depicts a schematic of the overall process from spectral acquisition to 3D image reconstruction.

## Results and discussion

**Figure 2:**
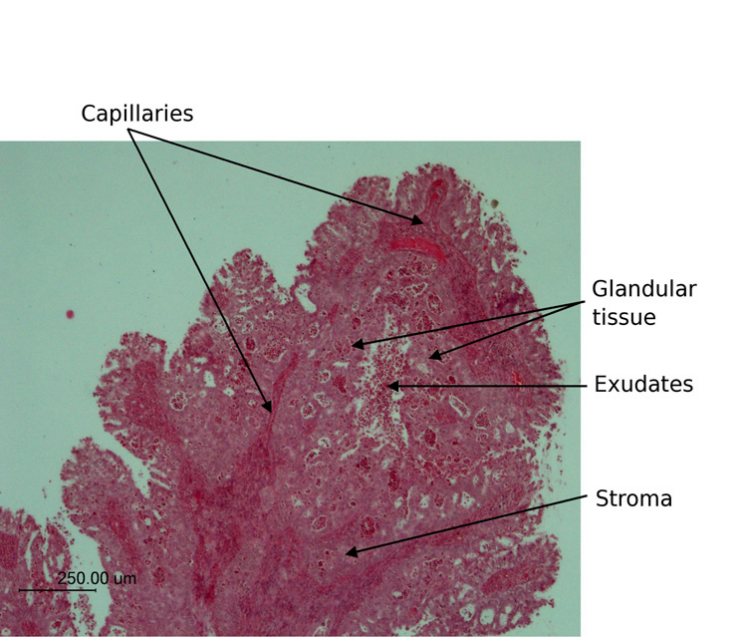
Light micrograph of a labeled H&E stained cervical section exhibiting villoglandular adenocarcinoma.

**Figure 3:**
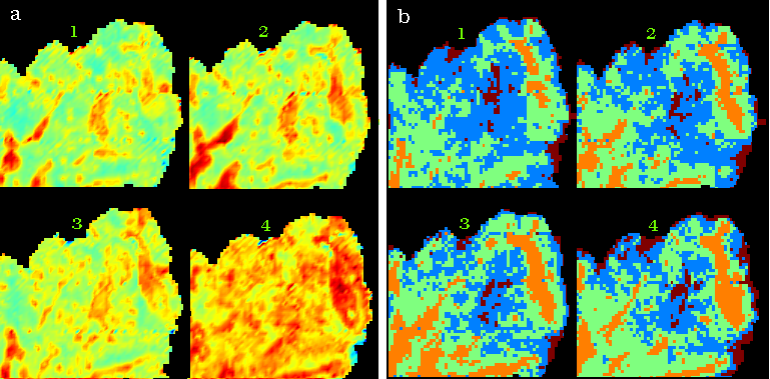
Hyper-spectral FTIR data processing performed simultaneously on 4 adjacent tissue sections from a cervical biopsy sample. The numbers 1 through 4 identify the individual sections in the figure. In (a), a univariate chemical image obtained from the integrating the area under the 1275-1190 cm^-1 ^region after baseline subtraction and in (b), a 4 cluster map derived from analysis over the 1272-950 cm^-1 ^spectral window. The cluster map false color scheme corresponds to brown for exudates, blue for inflamed glandular tissue, green for connective tissue and orange for blood filled capillaries as described in the text.

Figure 2 shows a H&E stained 2D cervical section exhibiting a relatively rare form of neoplasm known as villoglandular adenocarcinoma. The neoplasm is characterized by the presence of long villous fronds and papillae lined by columnar cells with intact cytoplasmic borders and displays minimal atypia. [25] Spherical clusters of cells with smooth intact communal cytoplasmic rings are also associated with this condition [25]. The sample makes an ideal model for 3D unsupervised hierarchical cluster analysis because it exhibits a variety of anatomical and histopathological features, including connective tissue, red blood cells, inflammatory exudate and glandular cells. Figure 3a depicts a chemical map generated from all four sections simultaneously by integrating the area under the band in 1275-1190 cm^-1 ^region associated mainly with phosphodiester contributions form nucleic acids. The chemical maps show a good correlation with morphology; however, specific correlations with anatomical and histopathological features cannot be gauged with this form of processing.

It is necessary to perform UHCA over the entire set of spectra collected to fully characterize the range of spectral variations through all the tissue sections. Performing separate UHCA on each individual tissue section would give a different clustering result due to changes (although generally small) in the biochemical composition between sections. For this reason the images were "stitched" together into a single frame to enable spectral pre-processing and UHCA to be performed in Cytospec™ on all spectra from all images simultaneously. UHCA was performed on the 1272-950 cm^-1 ^region on second derivative vector normalized spectra simultaneously on four adjacent sections and the resultant cluster maps are displayed in figure 3b. The cluster maps show a general similarity and successfully highlight the major anatomical features. The orange cluster represents red blood cells embedded in the stromal matrix. The light green cluster is predominantly stroma, while the brown is mainly lymphocyte exudates. The blue cluster is predominantly glandular tissue. In tissue sections 3 and 4 there is an increase in the area of connective tissue (green cluster) relative to glandular tissue (blue cluster) when compared to sections 1 and 2 indicating penetration of the glandular tissue into the connective layer.

**Figure 4:**
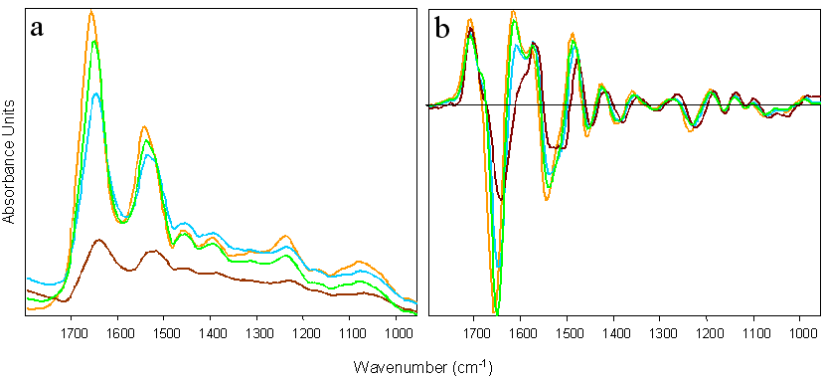
Mean cluster spectra (a) and mean 2^nd ^derivative cluster spectra (b) from a four cluster analyses based on the range 1272-950 cm^-1^and color coded to correspond to the cluster areas depicted in figures 3 (b).

Figure 4 shows the raw and second derivative mean extracted spectra color coded the same as the clusters in figure 3b. The maps and corresponding spectra are very similar for each section indicating that the biochemistry between the adjacent sections is consistent. The spectra exhibit dramatic changes in the amide I mode both in terms of bandwidth and position. The peak center varies from approximately 1643 cm^-1 ^to 1659 cm^-1^. This variation is attributed to physical-chemical changes in the tissue matrix. Dramatic variation occurs in areas of thin tissue and on the periphery of tissue sections with the net result a shifting of the amide I mode along with a concomitant increase in the amide II/amide I ratio. This effect is clearly observed in the mean extracted spectrum from the brown cluster which shows the amide I mode appearing at 1643 cm^-1 ^and an amide II/amide I ratio that is much greater for this spectrum when compared to the other spectra. Such strong distortions and shifts in band shape were recently addressed by Romeo et al. [9] who reported a method to correct for the "dispersion artifact". To minimize correlations with physical information the analysis was carried out using the 1272-950 cm^-1 ^region, which omits the proteinaceous range (1720-1380 cm^-1^) that may be strongly distorted by the dispersion artifact. Spectra from lymphocyte exudates and glandular tissue are dominated by a band at ~1240 cm^-1 ^which is assigned to the asymmetric phosphodiester stretching vibration of nucleic acids. This band shows the most variation between all 4 mean extracted cluster spectra. The mean extracted spectrum from the stromal areas (light green) has contributions from collagen vibrations although the distinctive collagen triplet in the 1300-1200 cm^-1 ^cannot be observed due to infiltration by red blood cells, lymphocyte exudates and glandular tissue into the connective layer.

**Figure 5:**
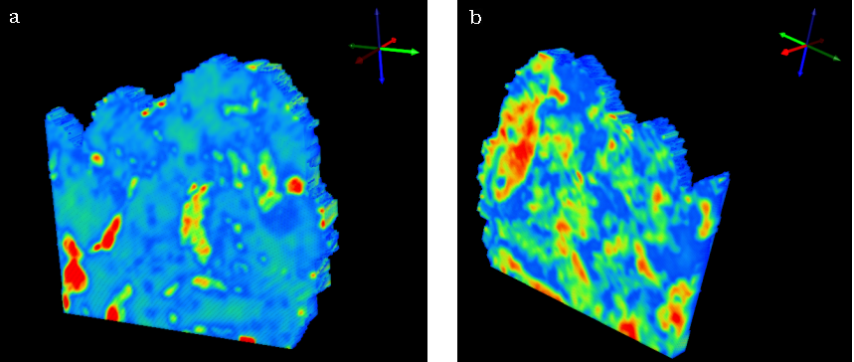
Two views of a 3D univariate chemical map plotting integrated absorbance over the spectral region 1275-1190 cm^-^1 after baseline correction. Red indicates areas of highest absorption and blue indicates areas of lowest absorption. The view is looking toward the section 1 side of the sampled volume in (a) and towards the section 4 side in (b).

The 3D chemical image constructed from 4 adjacent sections and generated by integrating the area underneath the peaks in the 1272-950 cm^-1 ^region is presented in figure 5. In figure 5a the image is orientated to show the first section of the tissue block (section 1) while in figure 5b the last section (section 4) is oriented towards the viewer. The darkest orange areas in section 1 (figure 5a) correlate well with the stroma and glandular tissue while the darkest orange area shown in section 4 (figure 5b) is associated mainly with the stroma.

**Figure 6:**
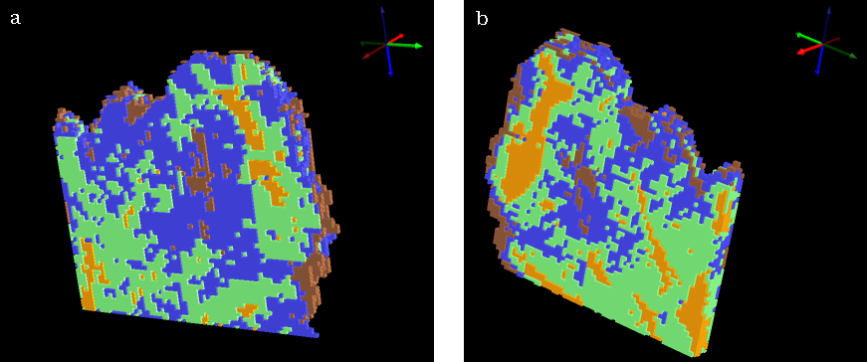
Two views of 3D cluster maps for 4 clusters obtained from analysis in the 1272-950 cm^-1 ^spectral region. The cluster map false colors are as described in the caption of figure 3 (b). The view is looking toward the section 1 side of the sampled volume in (a) and towards the section 4 side in (b).

**Figure 7:**
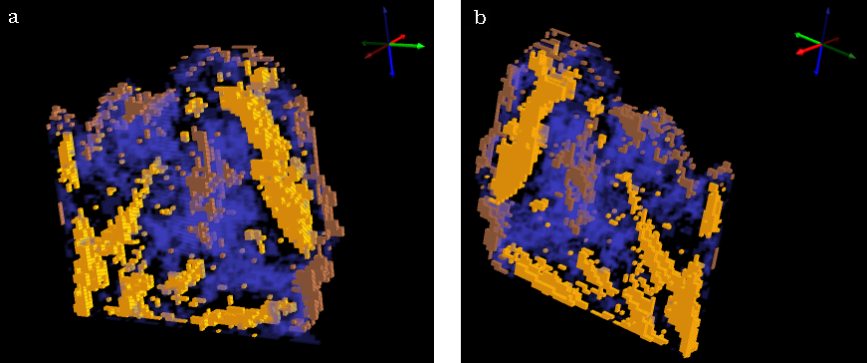
Two views of 3D cluster maps identical to the maps in figure 6 but with the stroma cluster removed from the plot and with glandular tissue now depicted in semi-transparent blue. The view is looking toward the section 1 side of the sampled volume in (a) and towards the section 4 side in (b).

Figure 6 shows a 3D UHCA map performed on the 4 sections simultaneously. The map shows excellent correlation with the anatomical and histopathological features indicated in figure 2. The cluster colors are the same as those used in figure 3b. The 3D UHCA map enables one to visualize the extent of penetration of the anatomical features and the degree of variation from section to section (see additional file 3: movie_adeno1.mpg for a rotating movie of this image). Moreover, 3D FTIR multivariate processing enables visualization of thick tissue sections that cannot normally be analyzed using conventional mid IR spectroscopic techniques due to the limited depth penetration of IR radiation. The thin sections (4 μm) required for use with the Kevley slides are less than the thickness of a single cervical cell consequently multiple sections enable the analysis of whole cells and also minimizes the effects of orientation artifacts that can arise during tissue sectioning. Individual clusters can be studied by rendering the image in semi-transparent mode. Figure 7 is identical to figure 6 but with the stroma cluster removed from the plot and with glandular tissue now depicted in semi-transparent blue. By making the image semi transparent one can visualize clusters in the center of the 3D FTIR image and examine the shape and penetration of important anatomical and histopathological features (see additional file 4: movie_adeno2.mpg for a rotating movie of this image).

The time required to acquire and compile 3D FTIR univariate (chemical) images involves the following intervals:

a) Approximately 10 minutes to acquire each 2D FTIR image from each individual tissue section.

b) Two minutes to run the MATLAB^® ^stitching program.

c) Registration of the images was done "by hand" in this study and was consequently quite time consuming, however, it is envisioned that suitable software could be developed to automate the registration process thereby reducing the time required for this to a few minutes.

d) About 1 minute is required for SCIRun to stack, interpolate and render a single 3D image frame.

A 3D univariate image could be obtained in less than 1 hour from commencement of FTIR scanning if a routine data-handling pipeline was incorporated. In approximately 1 hour a 3D movie, which are composed of a few hundred individual 3D image frames can be produced in SCIRun.

The production of 3D UHCA cluster images is a significantly slower process than generating univariate 3D maps because in addition to the steps delineated above for univariate maps UHCA must be performed. UHCA is computationally intensive and requires approximately 2 hours (Pentium 4, 3.4 GHz, Hyper-Threading, 2 Gb RAM) for the processing of four FTIR images stitched together. Compilation of UHCA 2D maps from large collections of tissue sections would be prohibitively slow for the current technique to have value as a rapid diagnostic tool. We are currently testing an artificial neural network alternative to UHCA.

FTIR imaging is resolution limited by diffraction to scales on the order of a few microns and hence, it is not always possible to unambiguously assign the obtained spectroscopic information as unique to particular sub-cellular structures. Nevertheless, FTIR imaging does provide valuable information on the overall biochemical composition when applied to tissue structures that are larger than a few microns in extent. The multivariate spectroscopic 3D-imaging method described in this work could be readily adapted for use with other emerging biophotonics techniques, most particularly Raman spectroscopic mapping which can provide macromolecular information on sub-cellular length scales.

## Conclusion

The coupling of vibrational spectroscopy with 3D multivariate processing greatly extends the capabilities of this technology in medical diagnostics. From a biomedical perspective existing pathological and histochemical protocols depend on sample morphology and visualization. Therefore the ability to maintain spatial integrity in three dimensions while assessing precise spectroscopic data intrinsic to a tissue sample represents an ideal combination. Three-dimensional multivariate processing provides a new way of visualizing tissue blocks based on the underlying biochemistry of the tissue matrix and will therefore have significant application in biology and medicine.

## Competing interests

The author(s) declare that they have no competing interests.

## Authors' contributions

BRW conceived of the study, and participated in its design and coordination and helped to draft the manuscript. KBR took the FTIR measurements and performed the image construction and helped draft the manuscript. CE developed the MATLAB^® ^routine for "stitching" multiple tissue sections. MQ provided the samples and did the histology. DM supervised the project and proof read the manuscript. All authors read and approved the final manuscript.

## Pre-publication history

The pre-publication history for this paper can be accessed here:



## Supplementary Material

Additional File 1 Proprietary MATLAB^® ^binary format. Program for stitching multiple tissue sections together for use in Cytospec™ spectroscopic software. Click here for file

Additional File 2 ASCII network file. File containing the 3D image processing program modules and configuration parameters for SCIRun. Click here for file

Additional File 3 MPEG movie. Rotating 3D cluster map of adenocarcinoma cervical tissue sample. Click here for file

Additional File 4 MPEG movie. Rotating 3D cluster map of adenocarcinoma cervical tissue sample with semi-transparent clusters. Click here for file
